# The Use of Vignettes to Improve the Validity of Qualitative Interviews for Realist Evaluation

**DOI:** 10.1177/10497323241237411

**Published:** 2024-03-19

**Authors:** Élisabeth Martin, Dave Bergeron, Isabelle Gaboury

**Affiliations:** 1 Département de médecine de Famille, 198734 Université de Sherbrooke, Longueuil, QC, Canada; 2 Département des sciences de la santé, 14846 Université du Québec à Rimouski, Rimouski, QC, Canada

**Keywords:** vignette, realist evaluation, interview

## Abstract

Although realist evaluation (RE) requires multiple data collection methods, qualitative interviews are considered most valuable and are most frequently used. The guiding principles of RE may limit the emergence of new Context–Mechanism–Outcome (CMO) configurations by evoking particular underlying mechanisms. This paper proposes a new method for conducting semi-structured interviews in the RE context by drawing on the literature and examining the ability of vignettes to explore perceptions about specific situations. Vignettes are developed based on researchers’ knowledge of the setting and program theory and are updated through an iterative process throughout data collection. Interviews focus on situations illustrated in the vignette to capture variations in interviewees’ perceptions. This method constrains interviewees to using retroduction to identify the hidden underlying mechanisms that link contextual elements to outcomes based on their experiences. This method allows researchers to focus on CMO configurations without evoking mechanisms, which contributes to the rigor of the method.

## Background

Realist evaluation (RE) is a theory-driven evaluative approach that has gained popularity in the past decade (G. Wong et al., 2016). RE aims to identify “what works under what circumstances and for whom” (Pawson & Sanjeev, 2009; Pawson & Tilley, 1997) and is a valuable approach to evaluate complex and constantly evolving interventions. The recent development of guides, recommendations, and tools has made the approach more accessible and contributed to its dissemination and utilization (e.g., Fick & Muhajarine, 2019; Manzano, 2016; Wong et al., 2016).

The realist approach aims to refine a program theory by identifying relationships between contexts, underlying mechanisms, and outcomes (Pawson & Tilley, 1997). However, although these are key concepts in RE, conceptual ambiguities about their definitions persist (Lemire et al., 2020; Nielsen et al., 2021). In this article, context refers to all pre-existing elements (de Souza, 2013; Hewitt et al., 2012). Depending on the focus, context can include observable features or relational and dynamic features (Greenhalgh & Manzano, 2022). The mechanism is an invisible element that can be triggered in a context to cause the outcome (Astbury & Leeuw, 2010; Hewitt et al., 2012; Lacouture et al., 2015; Westhorp, 2018). Mechanisms can include a specific program element, reactions to the program, or reactions to the context or a change in context (Lemire et al., 2020). Finally, the outcome is the result of one or more mechanisms activated following an intervention in a context (Lacouture et al., 2015; Pawson & Sanjeev, 2009; Ridde et al., 2012; Robert & Ridde, 2014). Context–Mechanism–Outcome (CMO) configurations conceptually link a context, mechanism, and outcome (or combination) by emphasizing the causal link between these elements (Ridde et al., 2012; Westhorp, 2018). Recurrent patterns of CMO configurations allow for the identification of regularity or demi-regularity to refine a program theory (Wong et al., 2016).

Realist evaluation often requires several types of data, but the qualitative interview remains the most common mode of data collection allowing for the emergence of mechanisms (Mukumbang et al., 2019). Qualitative interview techniques for RE have been specifically developed to improve usefulness and ensure rigor (Manzano, 2016; Wong et al., 2016). However, effectively eliciting CMO configurations in realist interviews without influencing interviewees’ perceptions remains a challenge. Traditional realist interviewing usually requires the directive wording of mechanism questions and a great deal of interview control (Bonell et al., 2022; Manzano, 2016; Pawson, 1996). In addition, realist methodology and interviewing techniques are complex and require a thorough understanding, which can overwhelm novice users and create barriers to utilization (Emmel, 2013; Pawson & Tilley, 1997).

The use of vignettes promotes the democratization and rigor of realist interviewing. Vignettes have been used in at least two REs, with the authors reporting that the method allowed interviewees some distance from their personal situations. This allowed interviewees to take a step back in order to stimulate in-depth discussions that explicitly identified mechanisms (Ohly et al., 2019; Sriranjan et al., 2020). Thus, the use of vignettes makes it possible to combine both the direct interview method proposed by Manzano (2016) and Pawson (1996) with the more indirect method proposed by Sayer (2000). The vignette can reproduce similar emotions and reflections as being in an actual situation. Therefore, this method may reduce memory and anticipation biases by having interviewees respond to the vignette context rather than try to remember their thinking at a specific moment in the past. This is particularly valuable in the context of a retrospective study. Clear guidelines will help democratize this method and increase the uptake of RE.

This article proposes a step-by-step method to use vignettes in realist interviews (example in Box 1). Before exploring the method, the precepts of qualitative interviews for RE are briefly described, followed by a description of vignettes and guidance for qualitative interviewing.

**Box 1. Case Example**

**RE of the champion to implement organizational change in primary healthcare clinics**

The vignette was used as part of a qualitative, multiple case study using an RE approach to describe how champions can promote quality improvement projects. In recent years, significant investments have been made to support various organizational changes in primary care. Studies suggest that the presence of a champion, a member of the organization who promotes innovation, facilitates the implementation of change. However, a better understanding of the underlying characteristics and mechanisms of champions to understand why and how they facilitate the integration of organizational change is needed.

A multiple case study was conducted in primary care practices engaged in an organizational change process. To describe the role of the champion in adopting organizational change and explore the factors that make champions effective, semi-structured interviews were planned with champions and change stakeholders to assess the initial theory.

Data analysis revealed regularities in the relationships between contextual elements, the champion intervention, the underlying mechanisms, and champion-related outcomes. The relationships between these three elements were represented in the form of CMO configurations. Validation with stakeholders also took place. This process, used iteratively but not linearly, allowed for the development of a final theory explaining the champion’s role in adopting organizational changes in primary care.

## Realist Interview Principles

RE almost always involves qualitative methods such as individual interviews or focus groups (Palm & Hochmuth, 2020; Renmans & Castellano Pleguezuelo, 2022). However, realist interviews can be challenging due to their focus on understanding how and why an intervention or program works, as well as the underlying mechanisms driving the observed outcomes (Manzano, 2016; Mukumbang et al., 2019; Pawson, 1996). To uncover these mechanisms, researchers must be flexible, adaptable, and open to unexpected findings. Specific challenges of conducting realist interviews, documented in several studies, include selecting appropriate participants, developing interview questions, and analyzing interview data (Salter & Kothari, 2014; Wong et al., 2016). Overall, conducting realist interviews requires a high level of skill and expertise, as well as a commitment to understanding the complex nature of interventions or programs and the mechanisms driving their outcomes.

Pawson (1996) first proposed a realist interview technique comprising the teaching–learning and conceptual focusing function. However, Goicolea et al. (2015) proposed that the question must be inspired by the initial program theory, without naming the theory explicitly. Manzano (2016) developed a more detailed and operationalized method for realist interviews based on Pawson’s recommendations, suggesting tips and proposing fundamental principles, but did not address the critiques of Pawson’s proposition. As no other guidelines have been proposed, these remain the current standard.

Following two particular principles makes an interview relevant to RE (Manzano, 2016): (1) plan the study based on the program theory and (2) ask questions according to a realist approach. To plan a study based on the program theory, researchers start by interviewing participants who know the program of interest well. They can provide a big picture of the program theory and fill large knowledge gaps by presenting a broad vision. Then, the researchers interview participants with more specific experiences to help refine the program theory. Realist interviews are divided into three phases: theory gleaning, theory refinement, and theory consolidation interviews (Manzano, 2016).

### Phase 1: Theory Gleaning Interviews

This phase aims to explore how the intervention is expected to work (Manzano, 2016; Pawson & Tilley, 1997, 2001). According to the initial theory, researchers use this first phase to obtain relevant information to articulate a preliminary understanding of the case context and outcome. Then, they formulate hypotheses of how, why, and under what conditions the program will work. Interviewees include program designers or people with a general understanding of the case or of the program’s functioning (Leeuw, 2003).

### Phase 2: Theory Refinement Interviews

Once the researcher has a general view of the case and has applied a program theory, the next step is to obtain empirical data to confirm, falsify, or refine that theory (Pawson, 1996). During this step, data collection and interviews aim to explore the related observed context and implementation features. For example, the researcher could explore the mechanism using retroduction.

### Phase 3: Theory Consolidation Interviews

These interviews aim to clarify the program theory or obtain missing information (Mukumbang et al., 2019). They are guided by the specificities of individual cases, and from there, they can be applied to the general program (Manzano, 2016). The consolidated theory must reflect the perceptions of actors involved in the program, their experiences, and the relationships between the program and its context (Wynn & Williams, 2012). This final phase of interviewing may involve reinterviewing key actors to probe, confirm, or clarify targeted aspects of the theory (Mukumbang et al., 2019).

To follow the second principle, that questions be asked according to a realist approach, the interviewer should understand what is happening in the natural setting to ensure a conversational feel to the interview. The aim of the interview is to understand the program’s story, not the participant’s story. It is also a key tool to explore micro-events that contribute to refinement of the program theory. The exploration of these elements allows fragments of theories to emerge within CMO configurations, which can be compared to other fragments to refine the program theory. Experts suggest presenting the theory to interviewees, who then confirm, falsify, or improve the theory according to their perceptions. To avoid “directing the interview,” Manzano (2016) proposed testing multiple theories with participants. In this way, the interview guide addresses the program theory and rival explanations. Similarly, Mukumbang et al. (2019) suggested that respondents may simply accept a proposed theory, which may interfere with the emergence of new CMO configurations by evoking particular underlying mechanisms. The method proposed here avoids this issue.

## Retroduction in Realist Evaluation

Retroduction is a key concept in RE. It is the most suitable form of reasoning in realist research and evaluation, as it encourages theorizing and enables testing of hidden mechanisms (Jagosh, 2020; The RAMSES II Project, 2017b). “Retroduction entails the idea of going back from, below, or behind observed patterns or regularities to discover what produces them” (Lewis-Beck et al., 2004). Retroduction allows for a focus on the causal links in CMO configurations. Retroductive logic involves understanding the explanatory causes of observations to produce a theory (Jagosh, 2020; Manzano, 2016; The RAMSES II Project, 2017b). In RE, data must be collected and analyzed using retroductive logic combining inductive and deductive elements with creativity and intuition (The RAMSES II Project, 2017b). The deductive element aims to test the initial theory, whereas the inductive element is used to formulate a modified version of the program theory based on available data (DeCarlo, 2018). Program theory allows for the development of emerging hypotheses (in the form of CMO configurations) that are evaluated through a deductive process (Justin et al., 2022). Analyses require the researcher to use their judgment, experience, imagination, and creativity to construct a theory (The RAMSES II Project, 2017a).

## The Use of Vignettes in Qualitative Research

A vignette is a short description of a person, an object, or a hypothetical scenario that can be used to prompt detailed reflections and examine cognitive processes (Atzmüller & Steiner, 2010; Herskovits, 1950; Rossi & Nock, 1982). Vignettes have been used in many disciplines to collect data on individuals or teams through individual or group interviews or questionnaires (Barter & Renold, 1999; Hughes, 1998; Hughes & Huby, 2002). In qualitative research, vignettes have shown potential to deeply understand healthcare professionals’ experiences (Sheringham et al., 2021; Tremblay et al., 2022). Vignettes allow qualitative researchers to focus on specific elements and help reveal interviewees’ true thoughts about a situation by depersonalizing the focus (Finch, 1987; Gourlay et al., 2014; Tremblay et al., 2022). We propose to use vignettes in qualitative interviews while respecting the standards for semi-structured interviews described by Kvale (2007) and Kvale and Brinkmann (2015): preparing questions carefully, establishing a relationship of trust with participants, active listening, and clarifying answers during the interview.

## Qualitative Vignettes in Realist Evaluation

Based on the case example presented in Box 1, we describe the steps involved in using a vignette in qualitative interviews: (1) research problem and program theory; (2) construct and write vignette; (3) sample and adapt vignette; and (4) interview using the vignette.

### Step 1: Research Problem and Initial Program Theory

The first step in designing an RE is to identify or develop an initial theory. The RAMESES II Project outlined six questions to choose, develop, construct, or refine the initial program theory (Wong et al., 2013). The theory should describe how and why mechanisms generate an outcome according to the context and include at least one CMO configuration (Wong et al., 2017).

In the example in Box 2, critical interpretive synthesis was used to determine the potential contextual elements and underlying mechanisms of a champion who may affect the implementation of organizational changes (Dixon-Woods et al., 2006; Martin et al., 2023). The initial program theory included many contexts to consider, interventions made by the champion that seemed to influence change adoption in the past, and mechanisms that may explain the champion outcome. The initial program also included CMO configurations based on previous analytical work and the authors’ retroduction.

**Box 2. Examples of Initial Program Theory**

Here are a few examples of the initial program theories we tested as part of the project and the associated contexts (C), mechanisms (M), and outcomes (O):

1. Having a champion provide feedback during the change (C) enables the quick adjustment of actions (M), which promotes the adoption of new behavior (O).
2. A champion who shares their experiences with peers (C) fosters the team’s confidence in the change proposal (M), which reassures clinicians and increases their commitment to change (O).
3. A champion who mentors and responds to queries quickly (C) helps maintain momentum during a change (M), making it easier to adopt new behaviors (O).

### Step 2: Vignette Writing and Construction

Vignette development and utilization in qualitative research requires robust methodological guidance to ensure the vignette’s relevance (Tremblay et al., 2022). The vignette must promote cognitive and emotional engagement and stimulate real-life contextual reasoning (Farmer et al., 2006; Finch, 1987). This should provoke participants’ intuition and instinctive answers, as a well-constructed vignette allows access to interviewees’ natural reactions and supports deep sincerity (Hughes & Huby, 2002). In realist interviews, vignettes should be developed based on the program theory without including any underlying mechanisms to reflect the system-level reality. Thus, vignettes represent the researchers’ knowledge about the context, program elements, and expected outcomes. When creating, using, analyzing, and interpreting the vignette, particular care must be taken to ensure validity. Validity is enhanced by considering multiple perspectives on a situation (Porter, 2007). Therefore, it is essential to ensure that the perspectives of different types of participants are included and to enhance the vignette with these different perspectives throughout the data collection process.

In the example, the vignette was developed using the initial program theory and known contextual elements and outcomes. Because vignettes differ from case to case depending on the context and outcomes, they evolve over time to represent the evolution of the initial program theory and the case. The vignette shown in Box 3 describes contextual elements (setting characteristics, change characteristics, setting elements, and the champion’s actions) and the outcomes (intermediate and final outcomes).

**Box 3.** Vignette

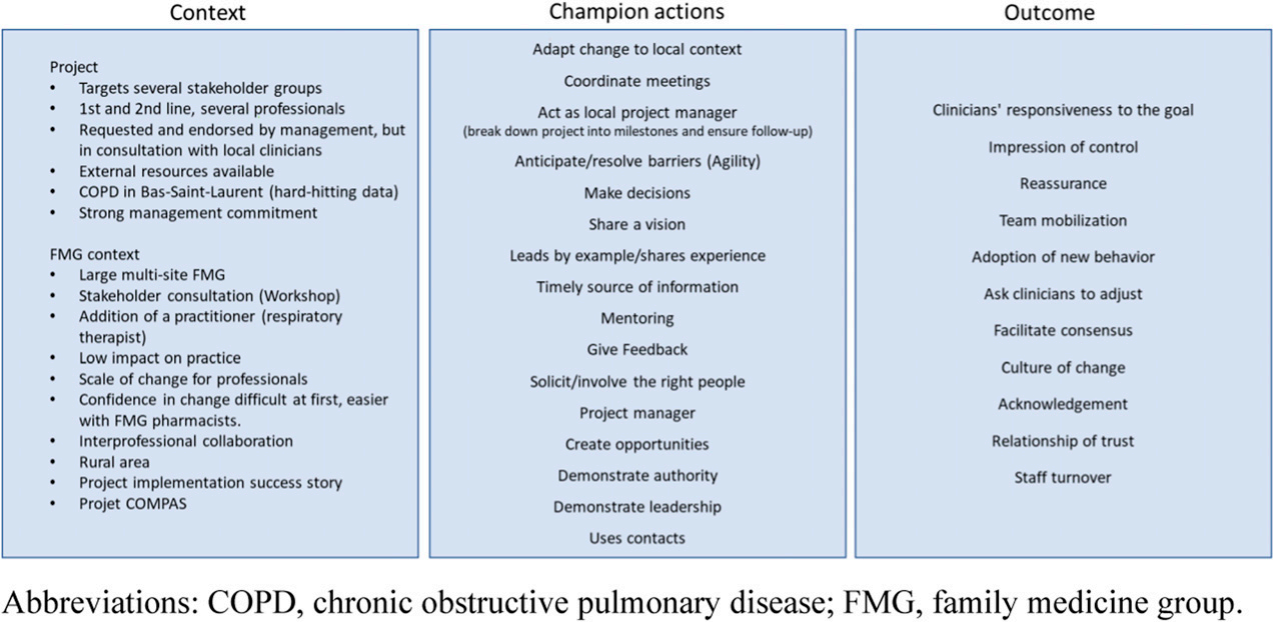

### Step 3: Sampling of Participants and Vignette Adaptation

For participant selection, it is advisable to follow the principles outlined by Manzano (2016) to interview those with a comprehensive understanding of the program or case under study. The more the researcher learns about the program theory and the specific case, the clearer and more supportive the vignette will be for the next interviewee. Further interviews with individuals who have directly experienced the program then provide insights into their reasoning and reactions to explain the information in the vignette. Ultimately, interviewees use the vignette to identify the mechanism linking the context and the outcome to finalize the CMO configuration. For example, the champion interview informed the researcher about the link between the intervention and context: “Which action did you take to facilitate the change implementation? Why did you take this action?” Then, peers made links between the champion’s actions and the outcomes using their own rationale and identified the causal mechanisms behind the link.

### Step 4: Interviewing Using the Vignette

Depending on the data collection phase (theory gleaning, refinement, and consolidation), the nature of the interviews is slightly different. Initially, interviews focus on better understanding the case. The researcher should begin by asking open-ended questions to discover, corroborate, or clarify relevant contextual elements, interventions, and outcomes. The researcher should actively listen to the interviewee and add relevant elements to the vignette. Then, the vignette, or parts of the vignette, is presented to the interviewee, with the interview guide focusing on specific situations illustrated by the vignette. Interviewees are then invited to add details they perceive as improving the vignette. This captures subtle variations among interviewee perceptions of the context and outcomes and gleans further knowledge about the case. Interviewees can then refine, validate, or correct the researcher’s analysis. The interviewer should then invite interviewees to link an outcome to a contextual or intervention element and explain their rationale, leading to the CMO configuration. In some cases, a cascade of mechanisms may be described, and the interviewer may need to ask for more information about the rationale. The interviewer can explicitly target a CMO configuration by asking interviewees to describe examples of linked elements and to complete the configuration by asking about the favorable context for that link. By exploring each micro-event, the interviewer can explore, gain more knowledge, and challenge elements.

In the case example (Box 1), interviewees clarified the context of specific elements of the organization and the change initiative (e.g., relations between members of the organization, perceptions of their peers, and the change initiative itself). They also described and added details about their feelings in general and during the change process. These elements are essential to build a holistic view of the context, intervention, and outcome. An example of an interview using the vignette is provided in Box 4. Probing helps to directly ask for mechanisms without specifying what the mechanism is. It can also provide some linked elements to help researchers understand or target specific configurations. The mechanism is the critical element of the RE approach. The vignette can also support case comparisons in a multiple case study performed using a realist approach. The interviewer could ask: “What do you think the outcome would be if [the champion] took this action in a larger organization?” Rather than presenting the theory or a rival theory, the mechanism is intentionally left to the interviewee. This method imposes the use of retroduction by the interviewee, thereby increasing the rigor of the data collection.

**Box 4. Examples of Questions and Prompts**

Interviewer: “Now I’d like to show you a vignette with three columns. In the first column, there is the context, in the second, the champion’s actions, and the last presents some potential effects of the champion’s actions. Each of these lists can be enhanced. The exercise is to link a champion’s action to an impact on implementation. Try to explain your rationale behind the link. I’ll leave you a few minutes to read and reflect upon them.”

Interviewee: “The champion shows authority. Clearly, I’m convinced that it reduces clinician receptiveness to the objective. If something is imposed, there’s less of a sense of belonging, less of a desire to ... In short, clearly, for me, authority reduces responsiveness.”

Interviewer: “And why?”

Interviewee: “Ah, but it’s clear that when something is more imposed there’s no sense of equality, and if someone is superior and asks you to do something it creates a bit of a break effect, to want to continue to implant yourself in something. That’s not what’s concerned you about it. And it’s clearly not soliciting a desire to prove yourself and then collaborate.”

In this example, the interviewer can also use other prompts to explore rival theories or nuances or to identify a more appropriate theory. For example, “Can you give me any contexts in which the use of authority might be helpful?” or “How does a sense of equality promote behavioral change?”

## Conclusion

An RE approach fosters better understanding of complex interventions and contributes a causal explanation of the observed outcome. However, this relatively new approach presents several challenges in its application. Tools and methods such as the vignette help democratize RE and improve its scientific rigor.

The method proposed here is based on the literature on vignettes and RE interview principles. It should contribute rich data on the causal explanations underlying observations made at the system level. Vignettes point out situations of interest in the same way Manzano (2016) has proposed. The difference is that, rather than presenting an underlying mechanism and asking participants what they think about it, the researcher asks participants to use reasoning to propose a mechanism. Vignettes encourage interviewees to make interconnections between contextual elements and outcomes, thereby improving the credibility of results and facilitating data analysis. However, as the number of interconnections increases, this method may require more interviews to saturate CMO configurations.

The realist interview remains challenging, but vignettes help to structure interviews and ensure rigor throughout the process.
